# Molecular Evolution of Functional Nucleic Acids with Chemical Modifications

**DOI:** 10.3390/molecules15085423

**Published:** 2010-08-09

**Authors:** Masayasu Kuwahara, Naoki Sugimoto

**Affiliations:** 1 Chemistry Laboratory of Artificial Biomolecules (CLAB), Graduate School of Engineering, Gunma University, 1-5-1 Tenjin-cho, Kiryu, Gunma 376-8515, Japan; 2 Frontier Institute for Biomolecular Engineering Research (FIBER) and Faculty of Frontiers of Innovative Research in Science and Technology (FIRST), Konan University, 7-1-20 Minatojima-Minamimachi, Chuo-Ku, Kobe 650-0047, Japan; E-Mail: sugimoto@konan-u.ac.jp (N.S.)

**Keywords:** chemical modification, random screening, post-SELEX, rational design, PCR, PEX

## Abstract

Nucleic acids are attractive materials for creating functional molecules that have applications as catalysts, specific binders, and molecular switches. Nucleic acids having such functions can be obtained by random screening, typically using *in vitro* selection methods. These methods have helped explore the potential abilities of nucleic acids and steadily contributed to their evolution, *i.e.*, creation of RNA/DNA enzymes, aptamers, and aptazymes. Chemical modification would be a key means to further increase their performance, e.g., expansion of function diversity, enhancement of activity, and improvement of biostability for biological use. Indeed, in the past two decades, random screening involving chemical modification, post-SELEX chemical modification, and rational design methods have been advanced, and combining and integrating these methods may produce a new class of functional nucleic acids. This review focuses on the effectiveness of chemical modifications on the evolution of nucleic acids as functional molecules and the outlook for related technologies.

## 1. Introduction

For living organisms, nucleic acids like DNA and RNA are fundamental biomacromolecules that function to preserve, transfer, and express genetic information. The potential of nucleic acids to behave as functional molecules had not attracted much interest due to the classic notions that proteins are functional molecules playing an important role in the body, whereas DNA is a blueprint of proteins and RNA is a mediator. However, in the 1980s, Cech *et al.* and Altman *et al.* discovered RNA enzymes (ribozymes) that catalyze RNA self-cleavage or RNA transesterification in splicing [1,2]. Since this discovery, much interest has been focused on the creation and application of functional nucleic acids with new activities that are different from the original activities of nucleic acids in the classical sense. 

Around 1990, Szostak *et al.*, Joyce *et al.*, and Gold *et al. *independently developed a screening methodology to select RNA molecules that can catalyze a specific reaction (ribozyme) or bind to a specific molecule (aptamer) from the RNA library (RNA pool of miscellaneous random sequences) [3,4,5]. This method is called *in vitro* selection; often called SELEX (systematic evolution of ligands by exponential enrichment) when used especially for screening aptamers. First, these screenings were performed for RNA, following which the creation of the DNA enzyme as catalyst and the DNA aptamer as specific binder was attempted using the *in vitro* selection method [6]. 

Single-stranded RNA/DNA enzymes and aptamers with a particular sequence, which were selected by the random screening methods, can exert their activities by forming a specific steric structure with intramolecular hydrogen bonding, stacking interactions, electrostatic interactions, and metal coordination. These RNA/DNA enzymes and aptamers can exhibit activity and functionality that are similar to those of protein enzymes and antibodies, respectively, though functional nucleic acids with activity superior to the corresponding protein have not been reported yet. Thus far, various RNA/DNA enzymes were reported, including the enzyme that catalyzes the isomerization of a bridged biphenyl and the one that catalyzes alkylation [7,8]. In addition, using *in vitro* selection, various RNA/DNA aptamers have been created, which are specific for a broad spectrum of targets involving small molecules like ATP or amino acids (e.g*.*, arginine), macromolecules like thrombin, and particles like Rous sarcoma virus (RSV) [9]. Furthermore, in the late 1990s, aptazyme (allosteric ribozyme) with allosteric activity was first developed by combining catalytic nucleic acids and aptamers [10]. At the beginning of the 21st century an interesting mechanism of RNA known as riboswitch was discovered in Nature [11]. 

However, some limitations on the ability of nucleic acid molecules to function as catalysts and specific binders were revealed. Although many examples of artificial molecular evolution of functional nucleic acids have been reported [12,13,14,15], the reason for these limitations is the fact that nucleic acids consist of a combination of only four nucleotides, while proteins consist of a combination of twenty amino acids with diverse functional groups in their side chains. Therefore, researchers have attempted screening by *in vitro* selection of modified nucleic acid enzymes and aptamers from chemically modified libraries, in which different functionalities that cannot be found in a nucleotide are incorporated. Using this process, RNA-cleaving enzymes and Diels–Alderases have been successfully created [16,17,18,19,20,21,22,23,24]*.* Among the modified nucleic acid enzymes reported, some enzymes that can catalyze *via* a reaction mechanism different from natural nucleic acids were found. 

Nucleic acid aptamers have attracted keen interest, especially for their potential medical uses. For applications in the medical field, improvement of nuclease resistance, *i.e.*, biostability in serum or cells as well as affinity to target molecules, became an important issue. An effective solution is screening of a specific binder from the modified nucleic acid library and the subsequent post-SELEX chemical modification of the selected aptamers. Regarding binding affinity, some modified nucleic acid aptamers can bind to proteins like enzymes or growth factors relative to a specific disease, with a dissociation constant of subnanomolar to subpicomolar, comparable to those reported for antibodies. Herein we describe and discuss the effectiveness of chemical modifications and the difficulties in developing functional modified nucleic acids.

## 2. Enzymatic Synthesis of Modified Nucleic Acids and Their Application to *in vitro* Selection

Random screening is a methodology used to obtain molecules that perform a desired activity, such as catalysis and specific binding, by displaying masses of diverse molecules called a library and selecting from them. This method involves phage display [25] and split–mix combichem [26] as well as *in vitro* selection. To evolve an *in vitro* selection method, that is, to adapt it to modified nucleic acids as well as to natural RNA and DNA, modified nucleic acids should be enzymatically synthesized by polymerase reactions. This is because the *in vitro* selection method would make good use of a unique feature of the DNA molecule that can be amplified and replicate its copy with a polymerase chain reaction (PCR). 

**Figure 1:**
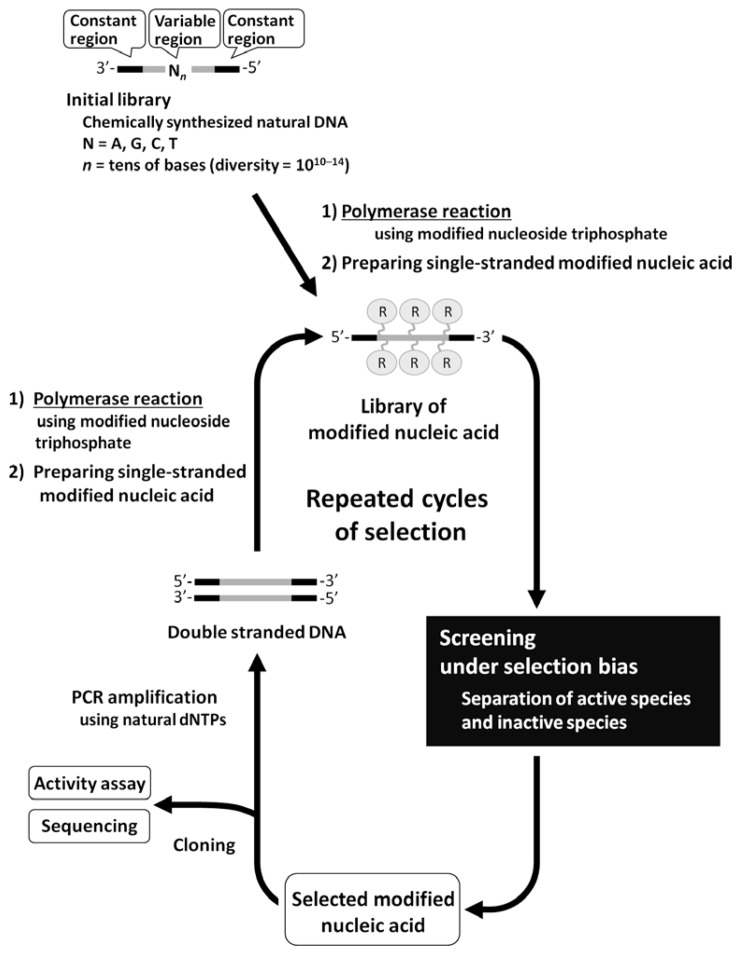
General scheme of *in vitro* selection using the library of modified RNA/DNA.

In cases of functional RNA screening, the process of PCR amplification can be implemented into the screening method, because the DNA is synthesized from an RNA template with reverse transcriptase, and the RNA is synthesized from a DNA template with RNA polymerase. Therefore, screening of functional modified nucleic acids could be performed by *in vitro *selection, if they can be directly amplified with PCR similar to DNA, or can be smoothly transcribed or reverse transcribed similar to RNA (Figure 1). However, yields of modified nucleic acids would not be sufficient for screening, or high misincorporation rates would occur if modified nucleoside triphosphates fail to function well as substrates for their enzymatic synthesis.

**Figure 2:**
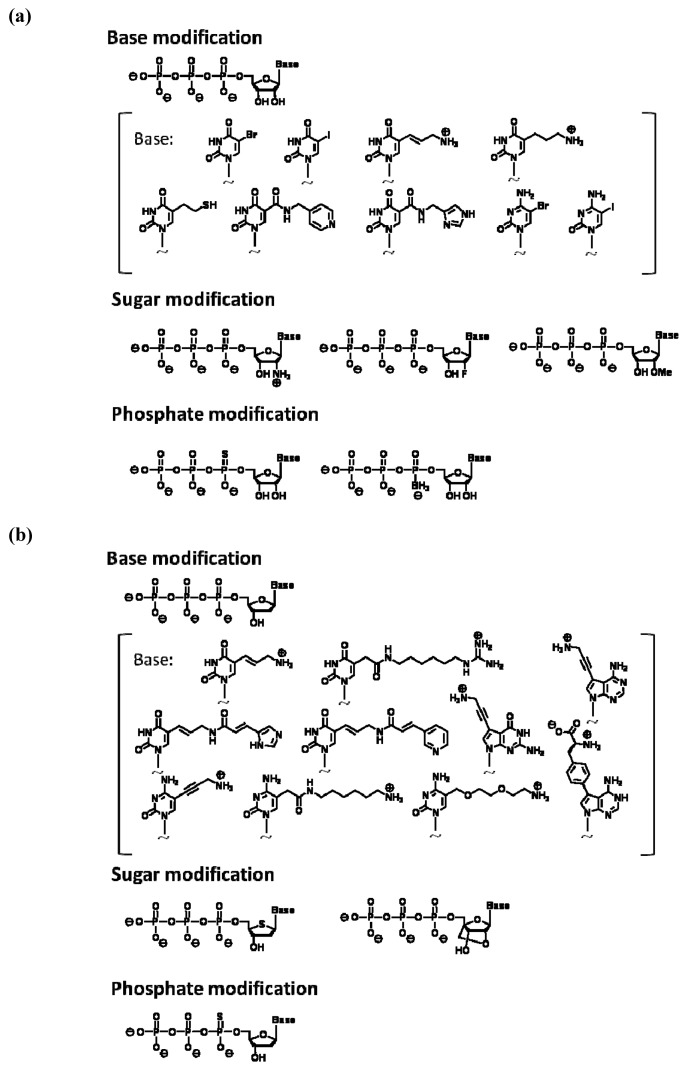
**(a)** Examples of modified nucleoside triphosphate analogs available for enzymatic RNA polymerization. **(b)** Examples of modified nucleoside triphosphate analogs available for polymerase chain reaction (PCR).

A monomer nucleic acid unit comprises base, sugar, and phosphate moieties, and each part could be an object of chemical modification. The following types of analogs are often used for *in vitro* selection using a modified nucleic acid library: pyrimidine analogs substituted at the 5 position and purine analogs substituted at the 7 or 8 positions of base moiety, analogs substituted at the 2' position of the sugar moiety, and phosphate analogs where oxygen is replaced with other chemical species. This is because the corresponding triphosphates of these analogs could act as relatively good substrates for a polymerase reaction. The production of modified nucleic acids would also be affected by the type of polymerase used, as well as the chemical structure and replaced position of the modified group. For modified RNA synthesis, the template DNA is transcribed using modified ribonucleoside triphosphates instead of natural triphosphates; T7 RNA polymerase was used in most reported examples, while SP6 RNA polymerase was also used. Figure 2a shows modified ribonucleoside triphosphates accepted as substrates by RNA polymerase [27,28,29]. 

Although these analogs are inferior to natural substrates with respect to incorporation efficiency, they can provide a relatively long strand of modified RNA as a full-length product corresponding to template DNA. Modified RNA synthesized especially using a uridine analog with various functionalities at 5 position, or uridine/cytidine analogs with a fluorine group or an amino group at 2' position, are often applied to *in vitro* selection. After screening, the selected modified RNA is reverse transcribed to DNA, and then amplified with PCR; natural nucleoside triphosphates are used as substrates in these processes. In the reverse transcription, the modified RNA is required to act as a template. Here, AMV (avian myeloblastosis virus) reverse transcriptase, *Tth* DNA polymerase with reverse transcription activity, or other types of modified RNA polymerases, are often used; the aforementioned modified RNAs were found to act as templates for the reaction catalyzed by those polymerases to yield the corresponding DNA products. 

For direct amplification of modified DNA with PCR, modified deoxynucleoside triphosphates are used as substrates. In PCR, modified substrates are needed to efficiently incorporate into the extending strand and the produced modified nucleotide strands are required to act as templates for the next thermal cycle. The incorporation efficiency is considerably dependent on the type of thermostable DNA polymerase used in PCR. Among the commercially available thermostable DNA polymerases for use in PCR, *KOD Dash*, *Vent(exo-)*, and *Phusion DNA* polymerases are known to be suitable. Our research data, which were obtained by scrutinizing misincorporation rates and successive incorporation of modified substrates, indicated that *KOD Dash* and mutants of *KOD *DNA polymerases derived from *Thermococcus *kodakaraensis **are the most suitable for modified DNA synthesis [30,31]. Moreover, *Taq* or *Tth DNA* polymerases that belong to the evolutional family A are found to be sensitive to chemical modification and not suitable for modified DNA synthesis. Thus far, deoxynucleoside triphosphate analogs of C5-substituted pyrimidine, C7-substituted 7-deaza-purine, and α-phosphoro-thioate have been known to function as good PCR substrates (Figure 2b) [30,31,32,33,34,35,36,37,38,39,40,41,42,43,44,45,46,47,48,49,50,51,52,53,54,55,56].

**Figure 3:**
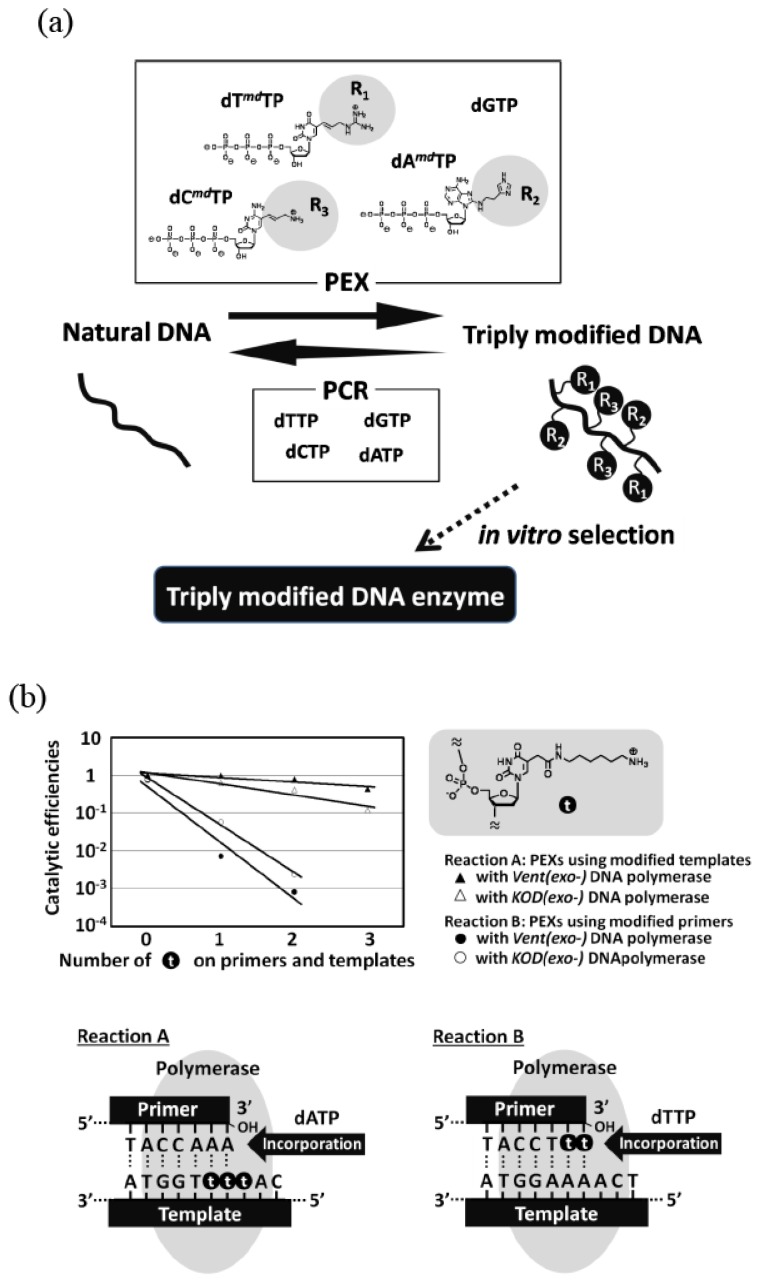
**(a)** Example of simultaneous incorporation of three different modified nucleoside triphosphates.In *in vitro* selection, a combination of PEX and PCR was used. **(b)** Effects of a modified group on nucleotide polymerization. The reaction B is far more inefficient than the reaction A.

Some modified triphosphate substrates considerably decrease the reaction efficiency and yield little or less amount of the modified DNA with PCR. In the selection using these analogs, the PCR amplification is performed using natural triphosphate substrates. First, the modified DNA is synthesized by a primer extension reaction (PEX) using the modified substrate and natural DNA template. After screening, the natural DNA is synthesized and amplified from the selected modified DNA as a template during PCR using a natural substrate. Then, the modified DNA can be obtained by transcribing the amplified DNA by PEX. The advantage of this method is that the modified DNA, into which many kinds of functionalities are incorporated, can be more efficiently synthesized compared with the aforementioned direct PCR amplification of modified DNA. This is because the scheme does not contain the simultaneous use of the modified triphosphate substrate and modified DNA template. Indeed, *in vitro* selections using libraries of doubly or triply modified DNA prepared by PEX were examined (Figure 3a) [16,17,18].

## 3. Chemically Modified RNA/DNA Enzymes

Various nucleic acid enzymes that catalyze reactions such as acylation, alkylation, and phosphorylation were artificially created by *in vitro* selection, while only two types of RNA enzymes that catalyze hydrolysis and transesterification were known in Nature, as described above. These types of catalysts were also obtained by screening from unique modified nucleic acid libraries. Examples of chemically modified RNA/DNA enzymes are listed on Table 1.

**Table 1:** Examples of chemically modified RNA/DNA enzymes.

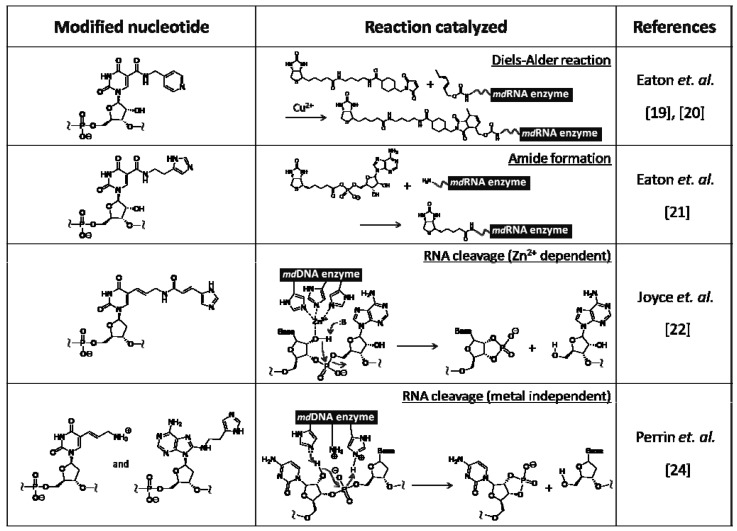

Eaton *et al.* created a modified RNA enzyme catalyzing the Diels–Alder reaction from a modified RNA library [19,20]. They used modified RNA containing uridine analog with a pyridiyl group at the 5 position instead of uridine. This RNA enzyme is considered to be able to exert its catalytic activity by coordination with a copper ion to the pyridiyl group, because divalent copper ion is required as a cofactor for catalyzing the Diels–Alder reaction. They also reported on another type of modified RNA enzyme that catalyze amide bond formation by adding biotinyl-AMP to an amino group tethered to the 5' terminus of RNA [21]. For this screening, the modified RNA containing uridine analog with imidazoyl group at the 5 position was used. The divalent copper ion is also necessary as a cofactor for catalysis. 

On the other hand, two examples of modified DNA enzymes, which catalyze cleavage of a particular sequence on RNA, are shown. In both cases, the modified DNA library was prepared by PCR amplification of natural DNA and subsequent PEX using modified triphosphate substrates. Joyce and Barbas *et al.* obtained a modified DNA enzyme catalyzing RNA cleavage under a zinc ion from a library of modified DNA containing a deoxyuridine analog with an imidazoyl group at the 5 position [22]. Joyce *et al.* had already reported a natural DNA enzyme with the same catalytic activity [23]. The activity of the modified DNA enzyme was found to be 10-fold higher than that of the natural DNA enzyme, while the catalytic domain of the former was 12 bases, which is smaller than that of the latter. These results indicate that the zinc ion, with which imidazoyl group is coordinated, could effectively work as catalyst.

Perrin *et al.* obtained a modified DNA enzyme from a library of doubly modified DNA involving both a deoxyuridine analog with allylamine at the 5 position and a deoxyadenosine analog with histamine at the 8 position, instead of the corresponding natural nucleotides [24]. Interestingly, the 20 bases of the modified DNA enzyme do not require metal ions for catalysis, and could cleave the RNA by acid-base catalysis of properly oriented amino and imidazoyl groups. The catalytic activity of these modified DNA enzymes was inferior to that of protein enzymes such as ribonucleases. The activity would be greatly improved by expanding the repertoire on catalytic functionalities. 

## 4. Chemically Modified RNA/DNA Aptamers

Chemically modified RNA/DNA aptamers were created mainly to enhance their nuclease resistance; examples were listed in Table 2. Modified RNA aptamers involving pyrimidine nucleosides modified with amino, fluorine, and methoxy groups at the 2' position were reported, because their triphosphate analogs are accepted as substrates for T7 RNA polymerase. Lin *et al.* obtained a modified RNA aptamer specific to the human neutrophil elastase (HNE) from a library of 2'-amino-modified RNA prepared with 2'-amino-UTP and 2'-amino-CTP instead of natural UTP and CTP [57]. The inhibition of HNE is expected to suppress various diseases to which the enzyme is related. The obtained modified RNA aptamer showed high affinity to target HNE with a dissociation constant (K_d_) of 7 nM. In serum, it was highly stable with a half life of approximately 20 hours, while natural RNA degraded within about 5 minutes. Modified RNA aptamers with other types of modification, *i.e.*, fluorine and methoxy groups, were created, and their improved nuclease resistance experimentally confirmed. Thus, these types of chemical modification were found to greatly improve the biostability of nucleic acids [27]. 

Regarding the effectiveness of the chemical modification to bind affinity and biostability, the following examples were reported. The modified RNA aptamer involving 2'-fluoropyrimidine nucleosides, specific for the Rous sarcoma virus, showed lower binding affinity but much higher stability than the natural RNA aptamer for the same target [58]. Pagratis *et al.* screened aptamers specific to the keratinocyte growth factor (KGF) from two libraries of modified RNA, which contained either 2'-fluoropyrimidine nucleosides or 2'-aminopyrimidine nucleosides, and compared both the aptamers [59]. In this case, the 2'-fluoro modified RNA aptamer showed excellent binding affinity with a dissociation constant (K_d_) of 0.3–3 pM, which was superior to the 2'-amino modified RNA. A modified RNA aptamer with both excellent nuclease resistance and high binding affinity was screened by *in vitro* selection. The 2'-amino modified RNA aptamer could be bound to basic fibroblast growth factor (bFGF) with high binding affinity (K_d_ = 0.35 nM), and was 1,000-fold times more stable in the presence of nucleases than the corresponding natural RNA aptamer; it was able to prevent bFGF from binding to its receptor on the cell surface at subnanomolar concentrations [60]. Except for the protein targets, the 2'-amino RNA aptamer, which was targeted to moenomycin A, was reported, it was found to be resistant for nucleases, but showed lower binding affinity (K_d_ = 300–400 nM) compared to that seen in modified RNA aptamers bound to proteins [61].

**Table 2:** Examples of chemically modified RNA/DNA aptamers.

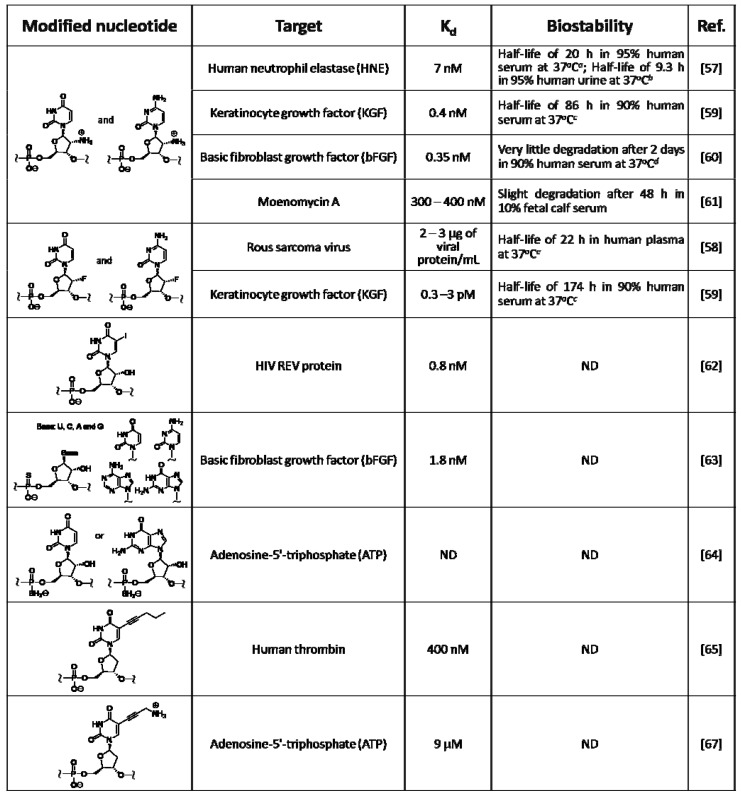

*^a ^*The half life of natural RNA was estimated as < 8 min; *^ b ^*The half life of natural RNA was estimated as < 4 min; *^ c ^*The half life of natural RNA was < 8 seconds; *^ d ^*The modified RNA aptamer was estimated to be at least 10^3^-fold more stable than natural RNA; *^ e ^*The modified RNA aptamer was estimated to be at least 10^2^-fold more stable than natural RNA. ND: not described.

Gold *et al.* obtained a base-modified RNA aptamer specific to the HIV REV protein, from the library of modified RNA containing 5-iodouridine instead of uridine; this base-modified aptamer could be bound to the target with somewhat higher binding affinity (K_d_ = 0.8 nM) compared to the corresponding natural RNA aptamer. Moreover, it was able to form a crosslink with the target protein by a UV irradiation process [62]. Few phosphate-modified RNA aptamers were made, because the T7 RNA polymerase could accept limited triphosphate analogs modified at phosphate moiety as substrate. Elligton *et al.* made a phosphate-modified RNA aptamer specific for bFGF by screening the library of modified RNA involving exclusively phosphorothioate internucleoside linkages, which could greatly enhance nuclease resistance. This aptamer was bound to bFGF with a binding affinity (K_d_ = 1.8 nM) approximately five times lower than that seen in the aforementioned 2'-amino RNA aptamer (K_d_ = 0.35 nM) [63]. As in the other example, the RNA aptamer involving boranophosphate, which is bound to ATP, was also reported [64].

**Figure 4:**
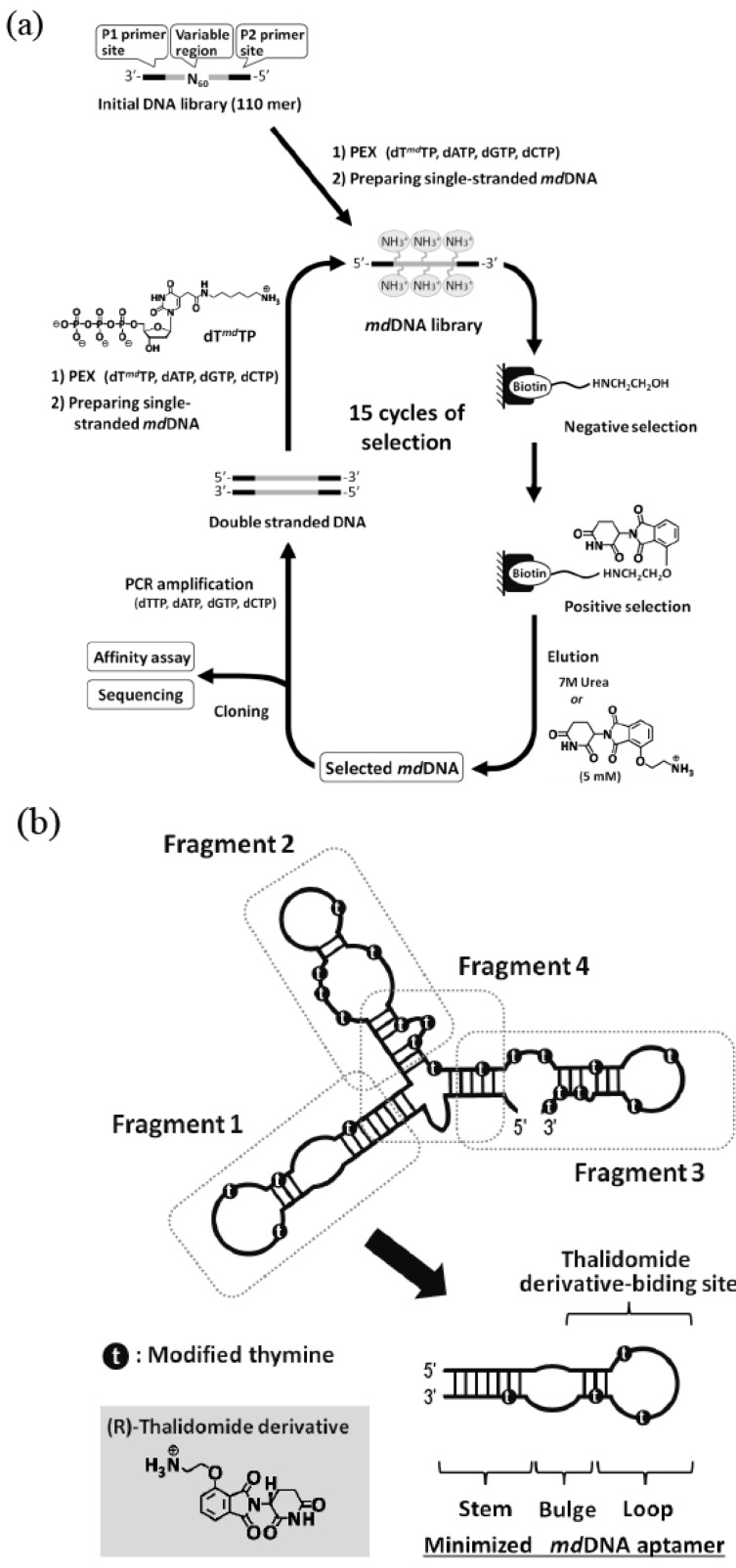
**(a)** Schematic illustration of *in vitro* selection of thalidomide-binding modified DNA aptamers. **(b)** Structure minimization of the obtained modified DNA aptamer based on secondary structure prediction.

Modified DNA aptamers made by *in vitro* selection were also reported. Lathman *et al.* obtained the modified DNA aptamer specific to the thrombin related to blood coagulation from the library of modified DNA containing 5-pentinyldeoxyuridine instead of thymidine [65]. The anti-thrombin aptamer is expected to be applied to an anticoagulant drug. To date, anti-thrombin aptamers have been obtained from libraries of natural RNA, natural DNA, and modified DNA [6,66]. The sequences of these aptamers were different from one another. The dissociation constants (K_d_) for thrombin binding ranged from 9.3 to 400 nM and the binding affinity increased in the following order; modified DNA < natural DNA < natural RNA. This order was the opposite in nuclease resistance. Benner *et al.* obtained a modified DNA aptamer specific to ATP from the library of cationic-modified DNA containing 5-aminopropynyldeoxyuridine [67]. The ATP-binding aptamers were also obtained from libraries of natural RNA and DNA [68,69]. Comparing obtained aptamers with the lowest K_d_ values, the binding affinity increased in the following order; modified DNA (9 μM) < natural DNA (6 μM) < natural RNA (0.7 μM). The modified DNA aptamer bound to ATP at a ratio of 1:2, while the natural DNA aptamer bound at a ratio of 1:1.

We obtained a modified DNA aptamer (K_d_ = 1 μM) specific to the thalidomide derivative from the library of modified DNA containing 5-(2-(6-aminohexylamino)-2-oxoethyldeoxyuridine. The modified DNA aptamer showed high enantioselectivity [70]. The detailed experimental studies on the effects of the modified group on binding affinity and specificity revealed that the modification was indispensable for the target binding (Figure 4). Such modified nucleic acid aptamers bound to medicines could be applied to drug delivery systems.

While chemical modification was shown to be remarkably effective in improving nuclease resistance, this superiority in modifying nucleic acids has not yet demonstrated the ability to enhance binding affinities. The correlation between the selection bias and the effectiveness of the modification in binding affinities should be further examined in detail in the future.

## 5. Post-SELEX Chemical Modification

Post-SELEX chemical modification is a method for heightening the performance of a RNA/DNA aptamer by introducing proper modification to the aptamer after *in vitro* selection using the natural nucleic acid library. For example, the following types of methods have been reported: replacing a phosphate moiety with phosphorothioate and capping the 5'-end with dialkyl glycerol [71,72]. Unfortunately, the modified RNA/DNA aptamers made by these post-SELEX methods generally showed lower affinities for the targets compared to the original unmodified aptamers.

The mirror-image aptamer, which was coined Spiegelmer, is one of the solutions for this issue. After screening RNA/DNA aptamers targeted to D-arginine, L-adenosine, or D-vasopressin, mirror-image aptamers with the same sequences as the selected aptamers were chemically synthesized using L-ribonucleotides or L-deoxyribonucleotides. These mirror-image aptamers have improved nuclease resistances, and can bind specifically to L-arginine, D-adenosine, or L-vasopressin [73,74,75]. 

The first aptamer drug, ‘Macugen’, which is recently approved to treat age-related macular degeneration (AMD), was made using Post-SELEX chemical modification [76]. Specific binders to Vascular Endothelial Growth Factor (VGEF) were screened from the library of modified RNA involving 2'-fluoropyrimidine nucleotides (U, C) and natural purine nucleotides (A, G). Then the natural A, G were properly replaced with 2'-methoxy nucleotides. In this process, to avoid affinity decrease and to enhance nuclease resistance, many modified oligonucleotides different in number and place from the replacement had to be synthesized and their performance evaluated.

If the chemical structures of the *in vitro* selected nucleic acid aptamer were altered as per requirements, or the modified nucleic acid aptamer were directly screened from nucleic acid libraries involving broad diverse modifications, then modified nucleic acid aptamers available for practical use could be smoothly created on demand. The former could be accomplished by developing rational design approaches, and then the latter could be done by developing rational random screening approaches.

## 6. Rational Design of Functional Nucleic Acids

The rational design approach is a way to create modified molecules with novel functions or improved performance by rationally designing their chemical structures based on data, of which important interactions in biomolecules were quantified. Fortunately, in the cases of RNA and DNA, their steric structure formations can be predicted almost correctly on the basis of the nearest-neighbor model [77]. This model is based on the concept that the duplex stability of nucleic acids is determined by the type of the neighboring base pair, but not by its sequence. However, regarding modified nucleic acids containing unnatural nucleotides altered at base-, sugar-, or phosphate-moieties, thermodynamic parameters concerning the effects of modified groups on stabilities of steric structures have not been investigated sufficiently, although new findings in the effects of modified bases and molecular environments are being obtained [78]. We expect to find a rule such as the nearest-neighbor model by carefully investigating correlations among structures, functions and stability of functional nucleic acids, and accumulating the relevant data. If the rational design of modified nucleic acids can be established, it would be possible to recreate high-performance modified aptamers from *in vitro* selected aptamers with ease [79].

## 7. Random Screening Methods without Repeated Cycles of Target Binding and Amplification

**Figure 5:**
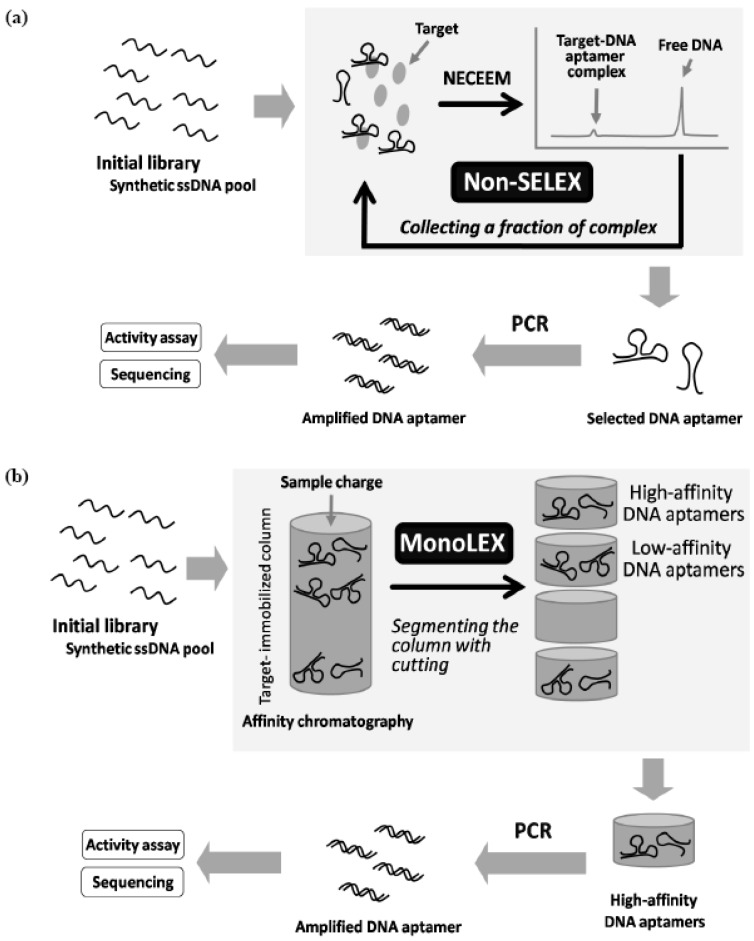
**(a)** Schematic illustration of random screening of DNA aptamers by non-SELEX selection. **(b)** Schematic illustration of MonoLEX random screening of DNA aptamers.

In random screening approaches with chemical modification, screening systems using libraries of unnatural nucleic acids involving broader types of modified groups should be constructed to diversify additional functionality, enhance activity, and improve biostability in biological use. One solution is to search for new polymerases or to develop genetically engineered mutants that are suitable for enzymatic-modified nucleic acid synthesis. Another solution is to develop an alternative screening system (Figure 5). 

For *in vitro* selection, it is necessary that both the following two reactions should proceed efficiently and accurately: 1) the reaction of the complimentary modified nucleic acid synthesis from DNA template, and 2) the reaction of the complimentary DNA production from the modified nucleic acid template. According to kinetic studies on polymerase reaction using modified nucleotides, the former reaction was found to be far more inefficient than the latter reaction (Figure 3b) [31]. Therefore, it can be understood that the former reaction is the most difficult pass in this screening system. 

**Figure 6:**
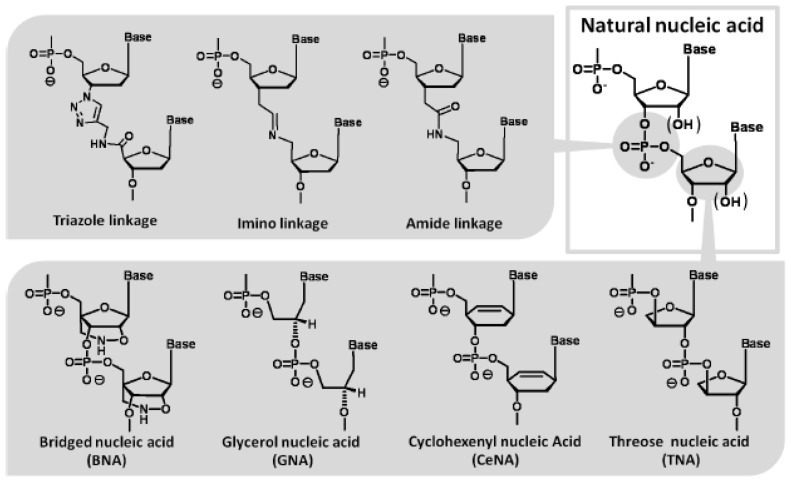
Modified nucleotides with unique chemical structures which act as a template in the polymerase reaction.

Recently, Berezovski *et al.* obtained DNA aptamers bound to MutS as a mismatch repair protein by a so-called non-SELEX selection using a nonequilibrium capillary electrophoresis of equilibrium mixtures (NECEEM) method [80]. This method is able to separate high-affinity aptamers from free low-affinity aptamers and non-binding nucleotides with high resolution. In addition, Nitsche *et al.* obtained DNA aptamers bound to vaccinia virus particles by a termed MonoLEX using affinity chromatography [81]. This method is effective in sorting high-affinity aptamers, low-affinity aptamers, and non-binding nucleotides. This method functions by segmenting the column with cutting, after loading the library on the target immobilized column and washing out with appropriate buffers. Both methods do not require repeated cycles of target binding and amplification in the screening system. Therefore, if these methods can be adapted to modified nucleic acid libraries, it would be possible to avoid the aforementioned most difficult pass, in principle. To date, polymerase reactions using modified nucleotides with unique chemical structures have been reported (Figure 6) [82,83,84,85,86,87,88]. This solution will make it possible to obtain functional nucleic acids from even libraries of these highly unnatural nucleic acids as with ease. 

## 8. Conclusions

Since the discovery of the RNA enzyme, researchers have investigated the possibility of using nucleic acids as functional molecules, such as catalysts, specific binders, and molecular switches. In those long-sustained efforts, researchers have attempted to use chemical modifications to obtain results such as the expansion of function, the enhancement of activity, and the improvement of biostability for biological use. Unfortunately, regarding enhancement of activity, no remarkable effects have been shown yet. However, regarding expansion of function and improvement of biostability, the research has clearly shown that chemical modification can be effective. Indeed, it is significant that a modified nucleic acid aptamer, which was made by SELEX and subsequent post-SELEX modification, can be practically used as therapeutic agent. The application range of modified functional nucleic acids can be very broad, because they have notable advantages that are not seen in protein enzymes and antibodies; for example, functional nucleic acids can be obtained by random screening without using laboratory animals and cultured cells, and they can be produced by organic synthesis at low cost. However, further technical innovations are required to bring the aforementioned performances close to those of functional proteins. By continuous development of random screening and rational design methodologies, we can expect that a new class of modified nucleic acid enzymes and aptamers with sufficient abilities for practical uses will be invented, and that these enzymes and aptamers will be suitable for applications to medical care, environmental analysis, food hygiene, and biological research.
